# Supplementary material for: Association between MGMT Enhancer Methylation and MGMT Promoter Methylation, MGMT Protein Expression, and Overall Survival in Glioblastoma
Source: Cells. 2023 Jun 15;12(12):1639. doi: 10.3390/cells12121639 (PMC10297309; doi:10.3390/cells12121639)
Supplement: Supplementary file 1 [file cells-12-01639-s001.zip › Supplementary_Figures.pdf]

Supplementary Figures

Association between *MGMT* Enhancer Methylation and *MGMT* Promoter Methylation, MGMT Protein Expression, and Overall Survival in Glioblastoma

Katja Zappe [1], Katharina Pühringer [1], Simon Pflug [1], Daniel Berger [1], Andreas Böhm [1], Sabine Spiegl-Kreinecker [2], and Margit Cichna-Markl [1,*]

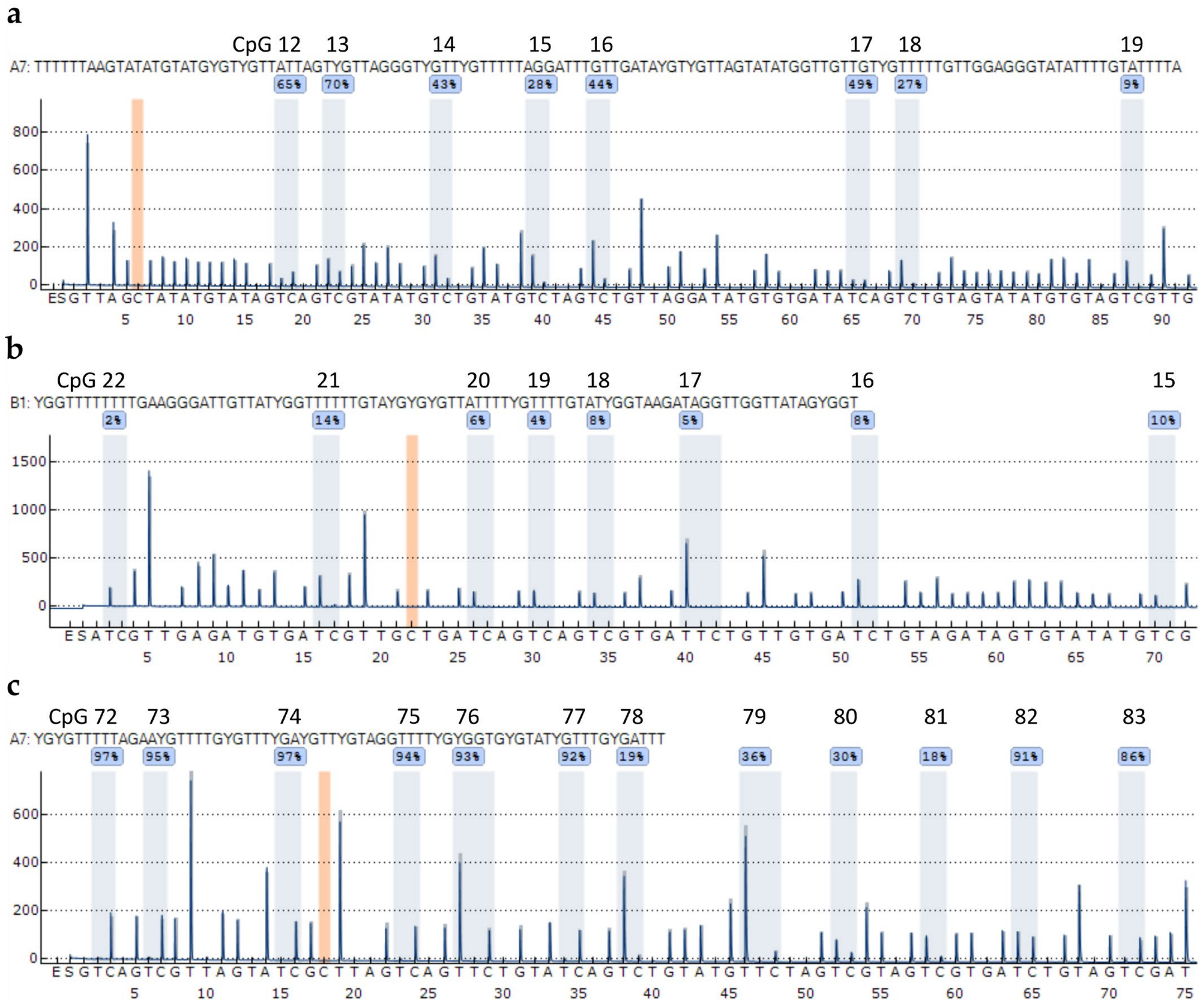

Figure S1. Representative pyrograms for (**a**) enhancer 1, (**b**) enhancer 3, and (**c**) the *MGMT* promoter for sample GBM08. Peaks highlighted in blue-grey indicate the position to analyze, grey bars represent the expected heights according to the dispensation order (histogram). Methylation levels of the CpG dinucleotides are shown above the respective position to analyze. Blue, yellow, and red colors indicate the quality of the result (blue: passed, yellow: check, red: failed). The position highlighted in orange allows for assessment of the efficiency of bisulfite conversion.

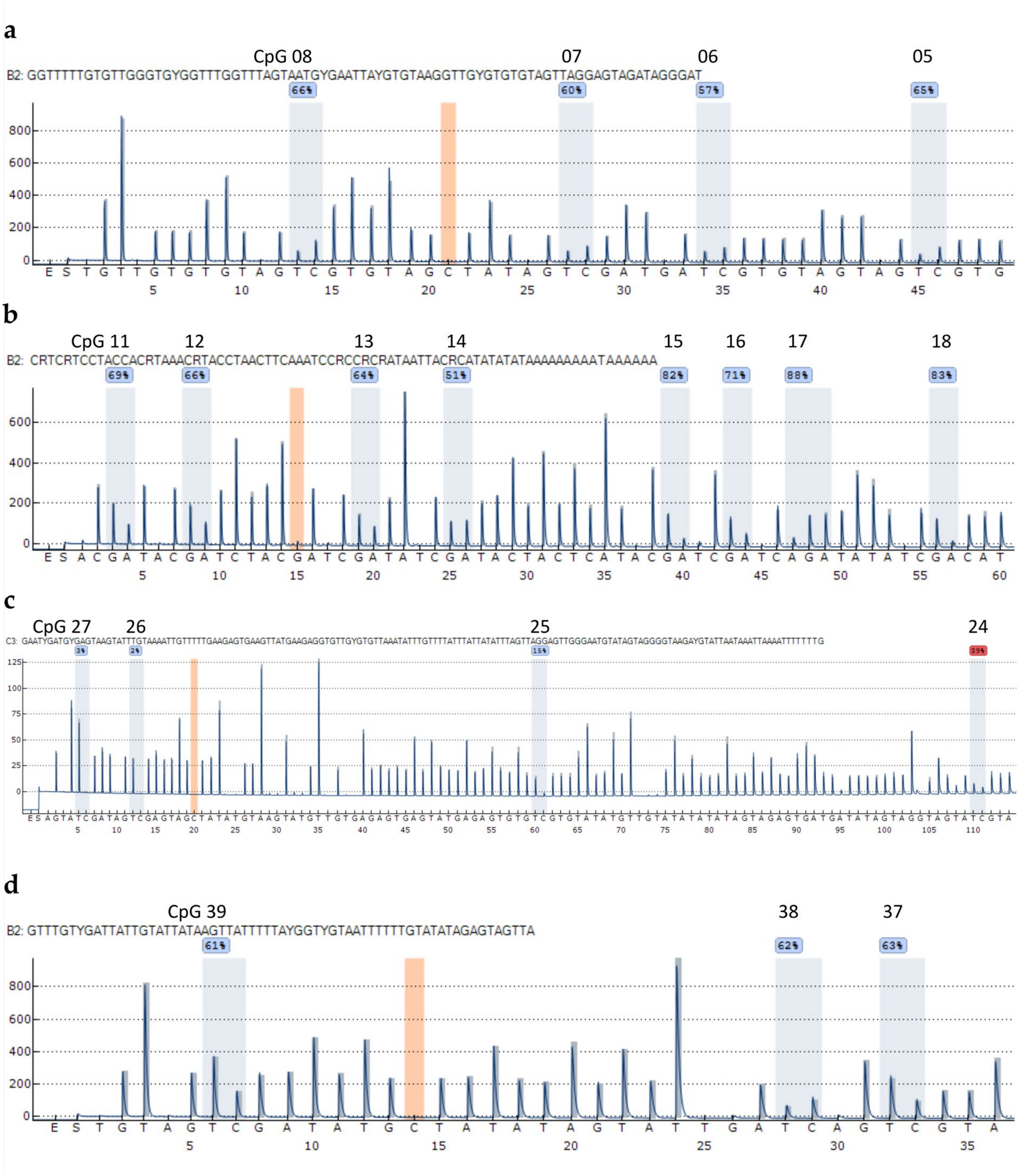

Figure S2. Representative pyrograms for enhancer 2 (**a**) assay A, (**b**) assay B, (**c**) assay C, and (**d**) assay D for sample GBM08. Peaks highlighted in blue-grey indicate the position to analyze, grey bars represent the expected heights according to the dispensation order (histogram). Methylation levels of the CpG dinucleotides are shown above the respective position to analyze. Blue, yellow, and red colors indicate the quality of the result (blue: passed, yellow: check, red: failed). Methylation levels of CpG 24 (assay C) were included into data analysis because results obtained for mixtures of methylated and non-methylated DNA standards confirmed that measurement was accurate in spite of failed quality flag. The position highlighted in orange allows for assessment of the efficiency of bisulfite conversion.

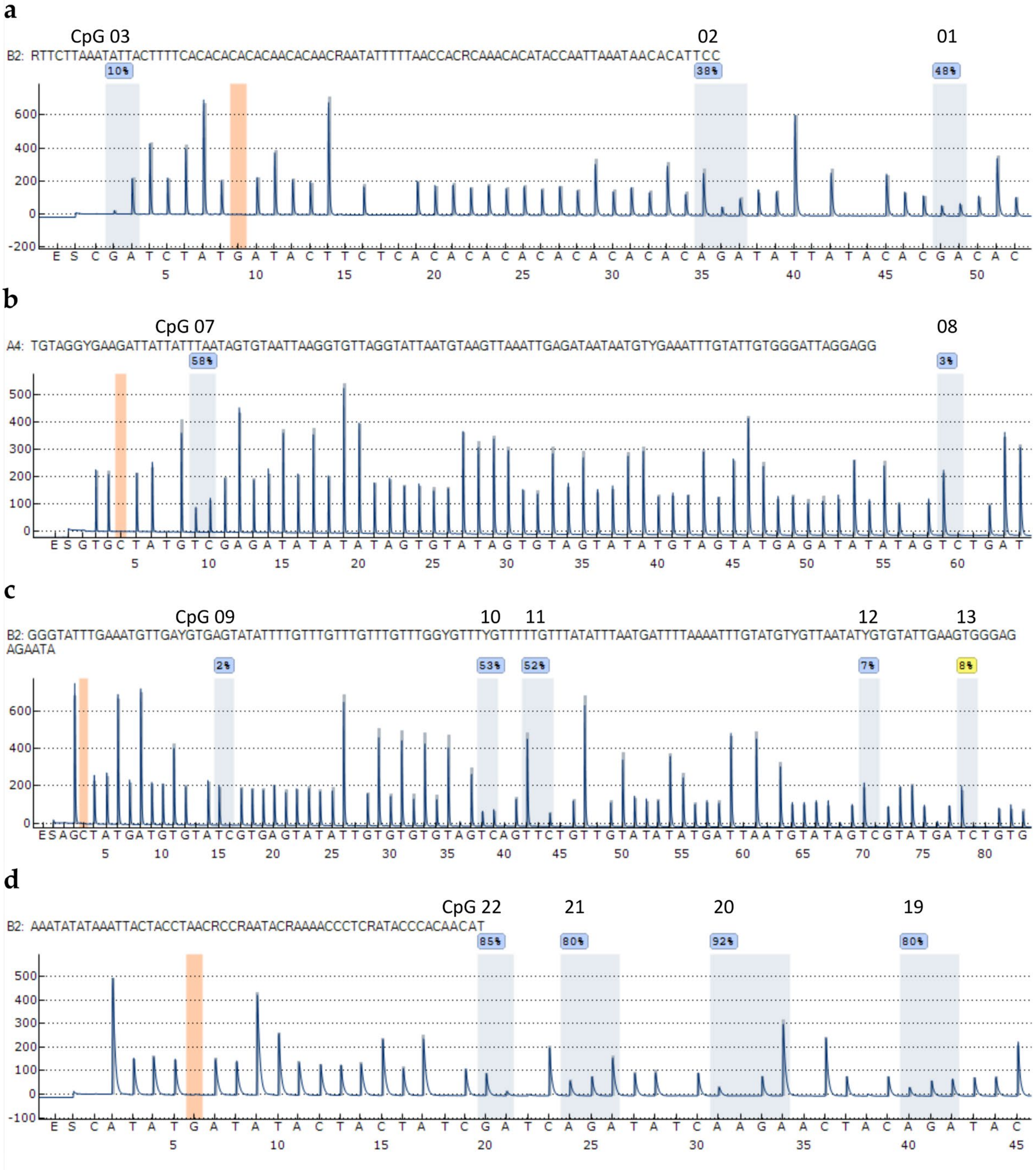

Figure S3. Representative pyrograms for enhancer 4 (**a**) assay A, (**b**) assay B, (**c**) assay C, and (**d**) assay D for sample GBM08. Peaks highlighted in blue-grey indicate the position to analyze, grey bars represent the expected heights according to the dispensation order (histogram). Methylation levels of the CpG dinucleotides are shown above the respective position to analyze. Blue, yellow, and red colors indicate the quality of the result (blue: passed, yellow: check, red: failed). The position highlighted in orange allows for assessment of the efficiency of bisulfite conversion.
