# Supplementary material for: Association between MGMT Enhancer Methylation and MGMT Promoter Methylation, MGMT Protein Expression, and Overall Survival in Glioblastoma
Source: Cells. 2023 Jun 15;12(12):1639. doi: 10.3390/cells12121639 (PMC10297309; doi:10.3390/cells12121639)
Supplement: Supplementary file 1 [file cells-12-01639-s001.zip › Supplementary_R-packages.pdf]

Supplementary R-packages

Association between *MGMT* Enhancer Methylation and *MGMT* Promoter Methylation, MGMT Protein Expression, and Overall Survival in Glioblastoma

Katja Zappe [1], Katharina Pühringer [1], Simon Pflug [1], Daniel Berger [1], Andreas Böhm [1], Sabine Spiegl-Kreinecker [2], and Margit Cichna-Markl [1,*]

David Robinson, Alex Hayes and Simon Couch (2022). broom: Convert Statistical Objects into Tidy Tibbles. R package version 1.0.1. https://CRAN.R-project.org/package=broom

Taiyun Wei and Viliam Simko (2021corrplot: Visualization of a Correlation Matrix (Version 0.92). https://CRAN.R-project.org/package=corrplot

Winston Chang (2014). extrafont: Tools for Using Fonts. R package version 0.17. https://CRAN.R-project.org/package=extrafont

Hadley Wickham (2021). forcats: Tools for Working with Categorical Variables (Factors). R package version 0.5.1. https://CRAN.R-project.org/package=forcats

Luke Smith (2016). ggloop: Create 'ggplot2' Plots in a Loop. R package version 0.1.0. https://github.com/seasmith/ggloop

Hadley Wickham (2016). ggplot2: Elegant Graphics for Data Analysis. Springer-Verlag New York. https://ggplot2.tidyverse.org

Alboukadel Kassambara (2020). ggpubr: 'ggplot2' Based Publication Ready Plots. R package version 0.4.0. https://CRAN.R-project.org/package=ggpubr

Gregory R. Warnes, Ben Bolker and Thomas Lumley (2021). gtools: Various R Programming Tools. R package version 3.9.2. https://CRAN.R-project.org/package=gtools

Stefan Milton Bache and Hadley Wickham (2020). magrittr: A Forward-Pipe Operator for R. R package version 2.0.1. https://CRAN.R-project.org/package=magrittr

Henrik Bengtsson (2022). matrixStats: Functions that Apply to Rows and Columns of Matrices (and to Vectors). R package version 0.62.0. https://CRAN.R-project.org/package=matrixStats

Simon Wood (2017) Generalized Additive Models: an introduction with R (2nd edition), CRC, New York. https://CRAN.R-project.org/package=mgcv

Philipp Schauberger and Alexander Walker (2021). openxlsx: Read, Write and Edit xlsx Files. R package version 4.2.4. https://CRAN.R-project.org/package=openxlsx

Bill Venables and Kurt Hornik and Martin Maechler (2019). polynom: A Collection of Functions to Implement a Class for Univariate Polynomial Manipulations. R package version 1.4-0. https://CRAN.R-project.org/package=polynom

Alboukadel Kassambara (2021). rstatix: Pipe-Friendly Framework for Basic Statistical Tests. R package version 0.7.0. https://CRAN.R-project.org/package=rstatix

Hadley Wickham and Dana Seidel (2020). scales: Scale Functions for Visualization. R package version 1.1.1. https://CRAN.R-project.org/package=scales

signal developers (2013). signal: Signal processing. http://r-forge.r-project.org/projects/signal/

Hadley Wickham (2019). stringr: Simple, Consistent Wrappers for Common String Operations. R package version 1.4.0. https://CRAN.R-project.org/package=stringr

Terry Therneau (2022). A Package for Survival Analysis in R. R package version 3.4-0. https://CRAN.R-project.org/package=survival

Alboukadel Kassambara, Marcin Kosinski and Przemyslaw Biecek (2021). survminer: Drawing Survival Curves using 'ggplot2'. R package version 0.4.9. https://CRAN.R-project.org/package=survminer

Wickham et al., (2019). Welcome to the tidyverse. Journal of Open Source Software, 4(43), 1686. https://doi.org/10.21105/joss.01686

Simon Garnier, Noam Ross, Robert Rudis, Antônio P. Camargo, Marco Sciaini, and Cédric Scherer (2021). Rvision - Colorblind-Friendly Color Maps for R. R package version 0.6.2. https://sjmgarnier.github.io/viridis/
