# Supplementary material for: Association between MGMT Enhancer Methylation and MGMT Promoter Methylation, MGMT Protein Expression, and Overall Survival in Glioblastoma
Source: Cells. 2023 Jun 15;12(12):1639. doi: 10.3390/cells12121639 (PMC10297309; doi:10.3390/cells12121639)
Supplement: Supplementary file 1 [file cells-12-01639-s001.zip › Supplementary_Tables.pdf]

Supplementary Tables

Association between *MGMT* Enhancer Methylation and *MGMT* Promoter Methylation, MGMT Protein Expression, and Overall Survival in Glioblastoma

Katja Zappe [1], Katharina Pühringer [1], Simon Pflug [1], Daniel Berger [1], Andreas Böhm [1], Sabine Spiegl-Kreinecker [2], and Margit Cichna-Markl [1,*]

Table S1. Optimized PCR conditions.

primer set	PCR mix	primer conc.	initial activation	PCR program				final elongation
				denaturation	annealing	elongation	cycles	
enhancer 1	mix 1	400 nM	95.0°C, 5 min	94.0°C, 15 s	56.4°C, 30 s	68.0°C, 30 s	50	68.0°C, 10 min
enhancer 2(A)	mix 2	400 nM	95.0°C, 15 min	94.0°C, 10 s	59.0°C, 20 s	68.0°C, 20 s	50	68.0°C, 5 min
enhancer 2(B)	mix 2	400 nM	95.0°C, 15 min	94.0°C, 10 s	58.0°C, 20 s	68.0°C, 20 s	50	68.0°C, 5 min
enhancer 2(C)	mix 2	400 nM	95.0°C, 15 min	94.0°C, 10 s	58.0°C, 20 s	68.0°C, 20 s	50	68.0°C, 5 min
enhancer 2(D)	mix 2	400 nM	95.0°C, 15 min	94.0°C, 10 s	58.0°C, 20 s	68.0°C, 20 s	50	68.0°C, 5 min
enhancer 3	mix 1	400 nM	95.0°C, 5 min	94.0°C, 30 s	52.6°C, 30 s	72.0°C, 30 s	45	72.0°C, 10 min
enhancer 4(A)	mix 3	200 nM	95.0°C, 5 min	94.0°C, 15 s	53.6°C, 30 s	72.0°C, 30 s	50	72.0°C, 10 min
enhancer 4(B)	mix 3	200 nM	95.0°C, 5 min	94.0°C, 15 s	56.5°C, 30 s	68.0°C, 30 s	50	68.0°C, 10 min
enhancer 4(C)	mix 3	200 nM	95.0°C, 5 min	94.0°C, 15 s	54.4°C, 30 s	68.0°C, 30 s	50	68.0°C, 10 min
enhancer 4(D)	mix 3	400 nM	95.0°C, 5 min	94.0°C, 15 s	54.2°C, 30 s	72.0°C, 30 s	50	72.0°C, 10 min
promoter	mix 1	400 nM	95.0°C, 5 min	94.0°C, 30 s	56.0°C, 30 s	72.0°C, 30 s	45	72.0°C, 10 min

mix 1: 1x EpiTect HRM Master Mix (Qiagen) in RNase-free water
mix 2: 1x PCR master mix consisting of 2.5 U HotStarTaq DNA Polymerase (Qiagen) in 1x supplied PCR Buffer, 200 nM of each dNTP (PCR grade dNTP mix, Qiagen), and 1x EvaGreen dye (Biotium, USA) in RNase-free water
mix 3: 1x Type-it HRM PCR Master Mix (Qiagen) in RNase-free water
primer conc.: concentration of each primer

Table S2. Dispensation orders for PSQ.

primer set	dispensation order
enhancer 1	GTTAGCTATATGTATAGTCAGTCGTATATGTCTGTATGTCTAGTCTGTTAGGATATGTGTGA-TATCAGTCTGTAGTATATGTGTAGTCGTTG
enhancer 2(A)	TGTTGTGTGTAGTCGTGTAGCTATAGTCGATGATCGTGTAGTAGTCGTG
enhancer 2(B)	ACGATACGATCTACGATCGATATCGATACTACTCATACGATCGATCAGATATATCGACAT
enhancer 2(C)	AGTATCGATAGTCGAGTAGCTATATGTAAGTATGTTGTGAGAGTGAGTATGAGAG-TGTGTCGTGTATATGTTGTATATATATAGTAGAGTGATGATATAGTAGGTAGTATCGTA
enhancer 2(D)	TGTAGTCGATATGCTATATAGTATTGATCAGTCGTA
enhancer 3	ATCGTTGAGATGTGATCGTTGCTGATCAGTCAGTCGTGATTCTGTTGTGATCTGTAGATAGTGTATATGTCG
enhancer 4(A)	CGATCTATGATACTTCTCACACACACACACACACAGATATTATACACGACAC
enhancer 4(B)	GTGCTATGTCGAGATATATATAGTGTATAGTGTAGTATATGTAGTATGAGATATATAGTCTGAT
enhancer 4(C)	AGCTATGATGTGTATCGTGAGTATATTGTGTGTGTAGTCAGTTCTGTTGTATATATGAT-TAATGTATAGTCGTATGATCTGTG
enhancer 4(D)	CATATGATATACTACTATCGATCAGATATCAAGAACTACAGATAC
promoter	GTCAGTCGTTAGTATCGCTTAGTCAGTTCTGTATCAGTCTGTATGTTCTAGTCGTAGTCGTGATCTGTAGTCGAT
